# The Molecular and Physiological Effects of Protein-Derived Polyamines in the Intestine

**DOI:** 10.3390/nu12010197

**Published:** 2020-01-11

**Authors:** Anna F. Bekebrede, Jaap Keijer, Walter J. J. Gerrits, Vincent C. J. de Boer

**Affiliations:** 1 Human and Animal Physiology, Wageningen University and Research, 6708 WD Wageningen, The Netherlands; anna.bekebrede@wur.nl (A.F.B.); jaap.keijer@wur.nl (J.K.); 2 Animal Nutrition Group, Wageningen University and Research, 6708 WD Wageningen, The Netherlands; walter.gerrits@wur.nl

**Keywords:** protein, polyamines, intestine, metabolism, mitochondrial function, hypusine

## Abstract

Consumption of a high-protein diet increases protein entry into the colon. Colonic microbiota can ferment proteins, which results in the production of protein fermentation end-products, like polyamines. This review describes the effects of polyamines on biochemical, cellular and physiological processes, with a focus on the colon. Polyamines (mainly spermine, spermidine, putrescine and cadaverine) are involved in the regulation of protein translation and gene transcription. In this, the spermidine-derived hypusination modification of EIF5A plays an important role. In addition, polyamines regulate metabolic functions. Through hypusination of EIF5A, polyamines also regulate translation of mitochondrial proteins, thereby increasing their expression. They can also induce mitophagy through various pathways, which helps to remove damaged organelles and improves cell survival. In addition, polyamines increase mitochondrial substrate oxidation by increasing mitochondrial Ca^2+^-levels. Putrescine can even serve as an energy source for enterocytes in the small intestine. By regulating the formation of the mitochondrial permeability transition pore, polyamines help maintain mitochondrial membrane integrity. However, their catabolism may also reduce metabolic functions by depleting intracellular acetyl-CoA levels, or through production of toxic by-products. Lastly, polyamines support gut physiology, by supporting barrier function, inducing gut maturation and increasing longevity. Polyamines thus play many roles, and their impact is strongly tissue- and dose-dependent. However, whether diet-derived increases in colonic luminal polyamine levels also impact intestinal physiology has not been resolved yet.

## 1. Introduction

Intake of protein when consuming a ‘normal’ Western diet contributes to around 15% of total energy intake [1], of which 85%–95% is digested in the small intestine [2]. The undigested protein from the small intestine, together with endogenously generated protein, moves towards the colon [3]. Here, the protein can be utilized by the microbiota to support their function and survival. Interestingly, by metabolizing the undigested protein, microbiota form and secrete metabolites that also impact the host [4]. Among these protein-derived metabolites, most are small molecule metabolites, like hydrogen sulfide, branched-chain fatty acids, phenolic compounds and polyamines [4,5]. Increasing protein intake beyond ‘normal’ levels has been shown to be effective for weight-loss management as well as sports performance [6,7]. Although these benefits of higher protein intake are well documented, it is not completely understood what the physiological effects are of increased concentrations of undigested proteins in the human colon. In pig studies, it has been shown that high protein intake leads to increased levels of the metabolites derived from protein fermentation in the gut, which have been associated with negative effects on gut health [8,9]. Among the protein fermentation end-products, polyamines have attracted much interest, in part, because of their essential role in cell proliferation, as well as their roles in other cellular functions, like cell growth, mitochondrial metabolism and histone regulation [10,11,12]. Combined, it is clear that polyamines influence multiple aspects of normal physiology. In this review, we focus on the biochemical, cellular and physiological aspects of the role of polyamines in regulating metabolism and proliferation in cells, with a special focus on the effects within the colon.

## 2. Polyamine Synthesis and Catabolism

### 2.1. Bacterial Polyamine Production in the Colon Is Dictated by Microbiome, Diet and Host Factors 

**Figure 1:**
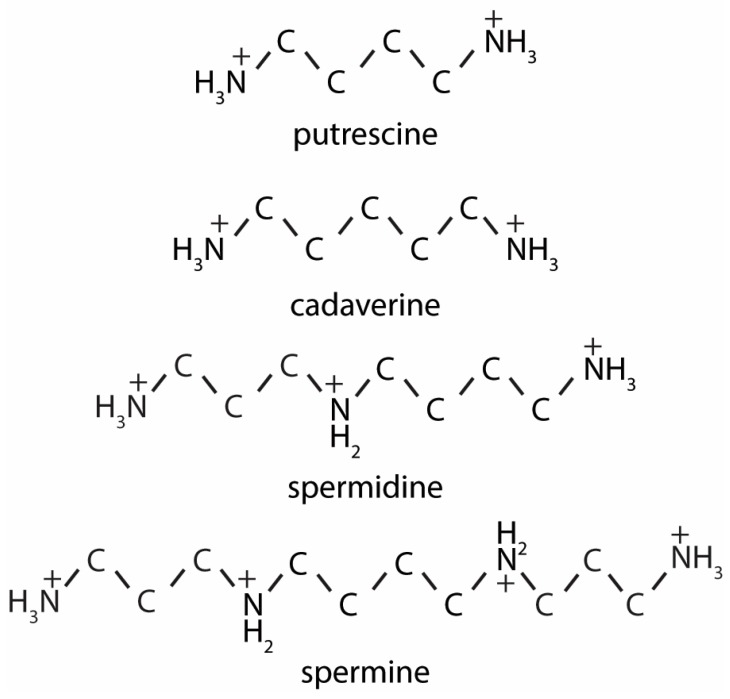
Polyamine structures. The two-dimensional structures of the diamines cadaverine and putrescine, the tri-amine spermidine and the tetra-amine spermine.

Spermine, spermidine, putrescine and cadaverine (Figure 1) are the most common polyamines in the human body. Based on fecal sample analysis it appears that putrescine is the most abundant polyamine in the human colon, followed by spermine, spermidine and cadaverine successively [13,14]. To produce polyamines, the microbiota in the colon need to break down proteins, in order to generate amino acids, which can serve as precursors for polyamine production [15]. Predominant bacterial species involved in proteolysis in the human gut are *Bacteroides* [16]. One resulting amino acid, arginine, can be converted, via citrulline, into L-ornithine, after which putrescine is produced [17]. Alternatively, arginine can also be converted into agmatine, which is then converted into putrescine, either directly by the enzyme agmatine ureaohydrolase, or indirectly through intermediate production of N-Carbamoyl-Putrescine [17]. Putrescine can then be further converted into spermidine and spermine. Recently, it was shown that microbial production of putrescine is a complex process, in which different bacterial species exchange polyamine intermediates, to finally produce putrescine [17]. The microbiome of humans mainly consists of the phyla Firmicutes (60%–80%) and Bacteroidetes (20%–40%) [18]. In other species, such as pigs, Firmicutes and Bacteroidetes are also the most abundant, but comprise a lower percentage of the total microbiome (~30% and ~10%, respectively) [19]. Although the contribution of these phyla to the total population of microbiota is lower in pigs, the functional metabolic pathways are similar between pigs and humans, indicating that microbial polyamine metabolism could be comparable [19].

Since microbiota produce polyamines from sources provided through the diet, dietary changes influence microbial polyamine production in the gut lumen. Indeed, in rats, diets high in fat were shown to increase spermine and spermidine concentrations in the mucosa, as compared to a soy protein-based diet, which led to much lower mucosal concentrations of these polyamines [20]. Similarly, in pigs that were given a feed containing casein, higher polyamine levels were found in the lumen of the proximal colon, as compared to the lumen of pigs fed a soy diet [21]. Since diets can shape the microbiome composition [22], the influence of diet on polyamine levels could also be explained by dietary-induced changing of the microbiota composition. This was for example shown for the proteolytic species *Bacteroides*, which become more abundant under conditions of higher protein availability in in vitro fermentations using human fecal inoculates [23]. This rise in proteolytic bacteria can thus contribute to increased polyamine levels in the colon by increasing substrate availability for polyamine production. Taken together, the most prominent determinants of luminal polyamine levels in an individual are found to be diet, host species and microbiome composition.

### 2.2. Mammalian Polyamines Synthesis Pathways 

**Figure 2:**
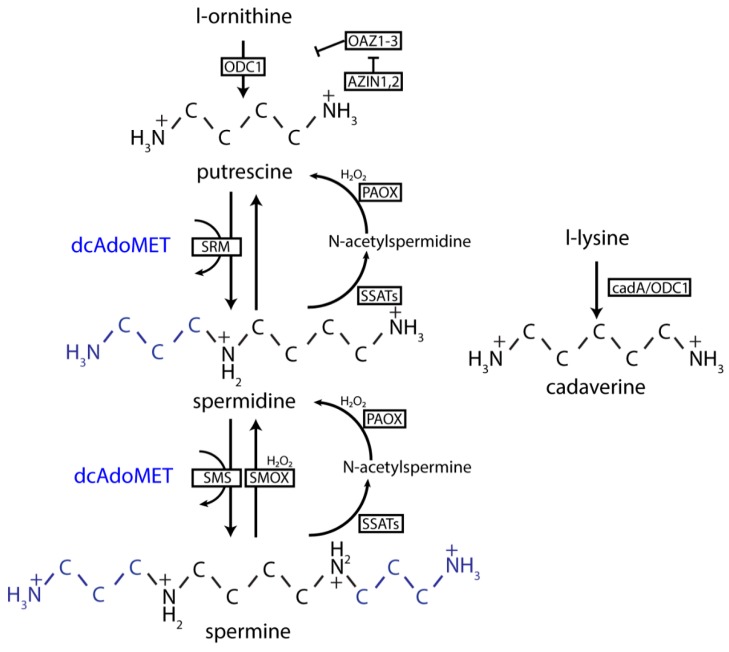
Polyamine metabolism. First, ornithine is converted into putrescine by ODC1. ODC1 is under tight control by OAZ1-3, which in turn is regulated by the antizyme inhibitor AZIN1-2. Together with dcAdoMET, putrescine can be converted into spermidine. Spermidine in turn can be converted into spermine, again with dcAdoMET as a co-factor. Both spermine and spermidine can be acetylated by SSAT. The acetylated product, but also spermine and spermidine themselves, can then be oxidized by PAOX. Both the acetylation and oxidation reactions produce reactive oxygen species (ROS). Cadaverine is synthesized though decarboxylation of lysine by bacterial cadA. ODC1: ornithine decarboxylase 1; dcAdoMET: s-adenosylmethioninamine; SSATs: spermidine/spermine N-1 acetyl transferases; PAOX: polyamine oxidase; cadA: inducable lysine decarboxylase.

In mammalian cells, the precursor for putrescine, spermidine and spermine is L-ornithine. Putrescine is synthesized via decarboxylation of the amino acid L-ornithine by the enzyme ornithine decarboxylase (ODC1) [12], which is one of the rate-limiting step in the formation of all downstream polyamines in this pathway. The diamine putrescine is converted to the triamine spermidine by spermidine synthase (SRM), and spermidine can be subsequently converted into spermine by spermine synthase (SMS) (Figure 2). SMS and SRM both use S-adenosylmethioninamine (dcAdoMET), the decarboxylation product of s-adenosyl methionine (SAM) as the aminopropyl-group source to produce spermidine and spermine. DcAdoMet is fully dedicated to the production of polyamines, since it is only used as an aminopropyl-donor for the formation of polyamines. Spermine can be converted back to spermidine through direct oxidation by spermine oxidase (SMOX) [24]. Alternatively, spermine can first undergo acetylation by spermidine/spermine-N1-acetyltransferases 1 and 2 (SSAT1, with a higher catalytic activity for spermidine; SSAT2, with equal catalytic activity for spermine and spermidine) before being oxidized by polyamine oxidase (PAOX) [12]. Acetylation by SSATs decreases the charge of polyamines, thereby making them less reactive and more easily degraded by PAOX [25]. Acetylspermine can also be oxidized by SMOX, but with a much lower K_m_ than spermine itself [25]. Although spermidine cannot be directly oxidized, it can be acetylated by SSATs, followed by oxidation by PAOX [26]. 

Cadaverine, a diamine like putrescine, is formed via a different route than the other polyamines. Cadaverine is synthesized from the amino acid L-lysine instead of from L-ornithine as for the other polyamines [27]. It is known that in bacteria, cyanobacteria and plants, L-lysine is converted into cadaverine by lysine decarboxylase (cadA), but in mammalian and fungal cells, the *cadA* gene has not been identified [28]. Cadaverine is nevertheless present in mammalian cells [29]. Therefore, some have suggested that cadaverine is also synthesized from L-lysine through ODC1 in mammalian cells. However, these findings come from experiments performed when both polyamines and serum are depleted [30], or in isolated tissues with very high ODC1 activity [31], but not under physiological conditions. In addition, even when blocking ODC1 activity, increased cadaverine levels were observed in cultured cells [32], suggesting that there may be other pathways involved in the mammalian production of cadaverine. Cadaverine can further be converted into its aminopropyl-form (aminopropylcadaverine), which is a close analogue of spermidine and may also be produced in mammalian cells via SRM [32]. 

## 3. Regulation of Polyamine Levels 

### 3.1. Regulation through Intracellular Polyamine Metabolism

Polyamine levels are tightly controlled within mammalian cells and several processes contribute to their regulation. Intracellular polyamine levels can be controlled through regulation of enzymes involved in polyamine metabolism. ODC1 protein levels are regulated by Antizyme (OAZ1-3), which forms a heterodimer with ODC1 and presents it to the 26S proteasome for ubiquitin-independent degradation [33]. OAZs themselves are regulated by the Antizyme inhibitor (AZIN1 and 2), which, amongst others, are homologous to ODC1 but lack its catalytic activity [34]. Under conditions of low polyamine levels, OAZs are repressed by AZINs, and ODC1 is synthesized. Synthesis of spermine and spermidine is further regulated by S-adenosylmethionine decarboxylase (AMD1), the enzyme that catalyzes the formation of dcAdoMet from SAM. Polyamines can actively regulate AMD1 expression and activity. AMD1 is transcribed as an inactive pro-enzyme, that undergoes auto-processing to turn into its active form [35]. Putrescine promotes autocatalytic cleavage of the AMD1 pro-enzyme, which leads to higher levels of active AMD1 in the cells, enabling the formation of spermine and spermidine [36]. In addition, putrescine can promote the catalytic activity of the enzyme by binding to the enzyme, which results in a electrostatic change that allows for improved conformation of the catalytic site [36]. Levels of spermine and spermidine are inversely correlated to levels of AMD1. Spermidine predominantly regulates transcription of the *AMD1* gene [37,38], while spermine seems to have a greater effect on the translation of the enzyme [38]. Furthermore, both spermine and spermidine influence the half-life of AMD1 [37]. 

The level of AMD1 is also regulated by the Mammalian target of rapamycin complex 1 (mTORC1), a protein complex generally promoting protein synthesis and growth [39]. The pro-enzyme form of AMD1 was shown to be phosphorylated by mTORC1. This prolonged the pro-enzyme’s half-life. Thus, phosphorylation of the pro-enzyme contributes to higher levels of the active enzyme [40]. This signifies that in a state of active growth and protein synthesis, mediated through activation of mTORC1, polyamine metabolism is upregulated. 

### 3.2. Regulation through Uptake and Transport

Polyamine levels are further regulated through uptake and export. While in prokaryotes uptake and export mechanisms have been described, little is known about these mechanisms in mammalian cells, despite continuous efforts to elucidate them. So far, no polyamine importer has been identified in mammalian cells. Instead, a caveolin-dependent endocytic uptake mechanism was discovered [41,42,43]. Polyamine export out of mammalian cells is mediated, at least in part, through SLC3A2, a member of the solute carrier family 3, that is involved in transport of L-type amino acids [44]. SLC3A2 is a diamine transporter that exports putrescine and imports arginine. However, interaction of this protein with SSAT suggests that it may also export acetylated polyamines [44].

## 4. Polyamines, Gene-Transcription and Proliferation

### 4.1. Polyamines Are the Substrates for the Post-Translational Modification Hypusine

A key role of polyamines is their ability to activate the eukaryotic translation initiation factor 5A (EIF5A) [45]. The name “eukaryotic translation initiation factor 5A” was given to the protein because initially it was thought that it acted as a translation initiation factor for all proteins. However, later it was discovered that EIF5A actually functions as a translation elongation factor [46,47]. Despite these findings, the protein is still known by its original name. Polyamines influence the activity of EIF5A via the post-translational modification of a specific lysine residue. Spermidine can be converted into hypusine (N-ε-(4-amino-2-hydroxybutyryl-lysine)) by deoxyhypusine synthase (DHPS) and deoxyhypusine hydroxylase (DOHH) in the cytosol [48]. Because of this modification, activated EIF5A is able to bind to ribosomes, enabling translation [46,47,49]. Since putrescine and spermine can both be converted into spermidine, these polyamines contribute to this process in a more indirect way, and the balance between them is dependent on specific cellular demands [50]. So far, EIF5A is the only protein found to have this hypusine post-translational modification. Indeed, the enzymes responsible for hypusination were shown to be EIF5A-specific [51,52,53]. Under physiological conditions, almost all the EIF5A protein present seems to be present in its active, hypusinated form (around 80%) [54]. 

### 4.2. Regulation of Eukaryotic Translation Initiation Factor 5A (EIF5A) Hypusination through Acetylation

EIF5A-hypusination was found to be regulated by acetylation of the hypusine residue by SSAT1 [55]. However, both spermine and spermidine are much better substrates for SSAT1, indicating that very little hypusine-acetylation will take place when sufficient levels of these polyamines are present [55]. Isolated acetylated hypusine-EIF5A from bovine testis, as well as recombinant acetylated hypusine-EIF5A, was shown to be inactive [55]. Thus, since hypusine-acetylation inactivates EIF5A, it seems that when there are insufficient levels of polyamines, EIF5A is inhibited and protein translation is decreased. Whether this mechanism also plays a role under physiological conditions remains to be seen, since intracellular polyamine levels are normally tightly controlled. However, it may prove an interesting therapeutic target under conditions where excessive cell proliferation occurs. 

### 4.3. EIF5A Hypusination Regulates Polyamine Synthesis 

A recent paper revealed how polyamine levels are regulated within the cells through hypusination of EIF5A [56]. The mechanism was shown to occur via AZIN1, the negative regulator of antizyme (OAZ1-3). Normally, translation starts when a 43S pre-initiation complex, consisting of a 40S small ribosomal subunit in combination with various initiation factors, encounters the start codon AUG. However, under certain circumstances, alternative start codons may be recognized, allowing for the transcription of alternative open reading frames [56,57]. In the presence of high polyamine levels (2 mM spermidine), ribosomes were shown to start queueing before the start of the main open reading frame (mORF), which lead to an increased start of translation at any weak start codon upstream of the mORF [56]. Start of translation at a weak start codon results in failure to properly translate and transcribe the AZIN1 protein, and thus repressed AZIN1 expression. As a result, OAZs are no longer inhibited by AZIN1. This leads to decreased synthesis of polyamines due to repression of ODC1 by OAZs. Thus, in this case polyamines cause stalling of ribosomes, instead of aiding translation elongation through EIF5A [56]. The authors state that high levels of polyamines may interfere with EIF5A hypusination, thus contributing to the stalling of ribosomes. It seems to be the case that optimal hypusination of EIF5A only takes place under low intracellular polyamine conditions, and thus only low concentrations of polyamines are needed to maintain maximal protein synthesis [58,59]. If this is the case, then increased intracellular concentrations may actually be detrimental for the cell.

## 5. Polyamines and Metabolic Functions

### 5.1. Polyamines Induce Mitochondrial Protein and Gene Transcription

**Figure 3:**
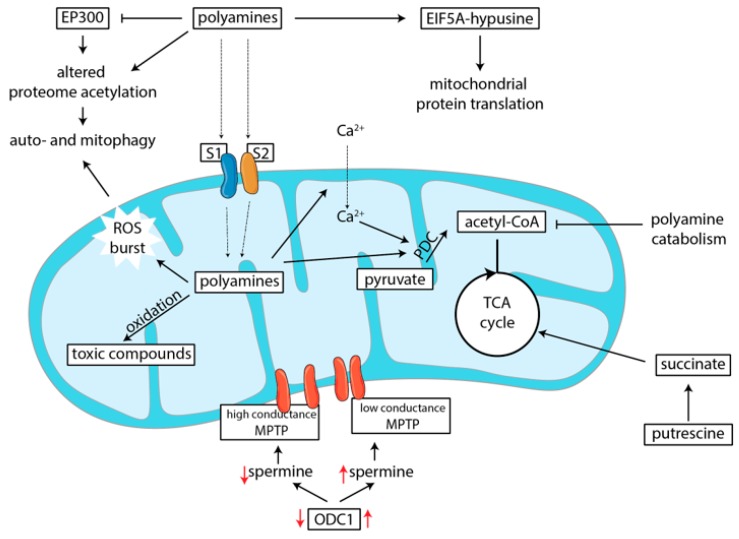
The role of polyamines in metabolism. Polyamines modify EIF5A by hypusination, which leads to upregulated of mitochondrial protein translation (Section 5.1). Polyamines can induce both auto- and mitophagy, through various mechanisms (Section 5.2). Firstly, through inhibition of the lysine acetyltransferase EP300, which leads to altered proteome acetylation and autophagy. Secondly, through induction of a ROS burst, which leads to activation of Ataxia-telangiectasia mutated (ATM) protein and more downstream induces mitophagy. Putrescine can, through conversion to succinate, serve as a direct energy source for small intestinal enterocytes (Section 5.3). However, polyamine catabolism can deplete acetyl-CoA levels, because it is used as a substrate by SSATs (Section 5.4). Polyamines are transported into mitochondria, where then influence respiration directly, through interaction with the pyruvate dehydrogenase complex (PDC), or indirectly by increasing Ca^2+^-concentrations, thereby increasing PDC activity (Section 5.5). At the same time, polyamine catabolism within the mitochondrial matrix leads to local production of toxic compounds, which may negatively affect respiratory functions (Section 5.6). But, polyamines can also protect mitochondrial membrane integrity through regulation of the mitochondrial permeability transition pore (Section 5.7). In red, regulation of spermine levels is indicated. When ODC1 is upregulated, more spermine is produces which induces the formation of the low conductance state of MPTP. This helps to maintain proper membrane integrity. When ODC1 activity is decreased, intracellular spermine concentrations are decreased, and the high conductance state of the MPTP cannot be prevented, resulting in mitochondrial swelling. EP300: E1A-binding protein p300; ROS: reactive oxygen species; MPTP: mitochondrial permeability transition pore; EIF5A: eukaryotic translation initiation factor 5A; TCA: tricarboxylic acid cycle; SSATs: spermidine/spermine N-1 acetyl transferases; S1 and S2: mitochondrial polyamine transporters 1 and 2.

The role of polyamines in transcription can specifically influence mitochondrial function. An overview of how this regulation of polyamines on metabolic function occurs is depicted in Figure 3. In macrophages, hypusination of EIF5A was found to induce transcription of mitochondrial genes (Figure 3). When macrophages are activated, they can either adapt a pro- or an anti-inflammatory phenotype, depending on cues from the environment. These phenotypic adaptations require, and are even driven by, metabolic changes within the cells [60]. Thus, alterations in metabolic function influence the phenotypic outcome of differentiation from monocyte to macrophage. Puleston et al. showed that hypusination of EIF5A plays a crucial role in the metabolic switch of macrophages. In the absence of hypusination of EIF5A, oxygen consumption, and thus mitochondrial function, was greatly reduced [61]. Because of this, macrophages could not adapt an anti-inflammatory phenotype, instead resorting to aerobic glycolysis, which resulted in differentiation of macrophages towards a more pro-inflammatory phenotype [61]. Another study showed that deletion of ODC1, and thus depletion of intracellular polyamine levels, led to a marked increase of pro-inflammatory macrophages [62]. As a result, increased stomach and colonic inflammation was observed. Here, the mechanism was attributed to the altered methylation status of the chromatin, which led to gene transcription that favored pro-inflammatory macrophage differentiation [62]. 

In kidney cells, the importance of EIF5A-hypusination on mitochondrial protein expression was also observed [63]. Melis et al. [63] showed that inhibition of hypusination by intraperitoneal injection of rats with the deoxyhypusine synthase inhibitor N1-guanyl-1,7-diaminoheptane (GC7) led to decreased expression of mitochondrial complexes. This correlated with lower mitochondrial respiration [63]. The decrease in mitochondrial respiration lead to lower reactive oxygen species (ROS) production during hypoxia. Thus, decreased hypusination could protect kidney cells from oxidative damage through downregulation of mitochondrial protein expression. Based on their observations, higher polyamine levels should thus lead to higher EIF5A hypusination levels, and also higher mitochondrial respiration in kidney cells. 

### 5.2. Polyamines Can Induce Mitophagy

Induction of mitophagy has been considered a mechanism to protect from cell death and can even be a mechanism to maintain and optimize metabolic function [64]. Indeed, in a study that examined mitochondria in heart muscle in hypertension and aging, spermidine was shown to induce mitophagy, thus removing damaged mitochondria, resulting in improved metabolic function and cell survival (Figure 3) [65]. This effect was also observed in neuronal cells where aging was induced [66]. Both mitophagy and autophagy can be induced through decreased mTORC1 activation. However, whether spermidine induces inhibition of mTORC1 is unclear, with some studies reporting inhibition of mTORC1, while others not observing this, even when using the same concentration of spermidine [67,68]. A consistent finding is that spermidine alters the proteome acetylation status [67,68,69], at least in part through inhibition of E1A-binding protein p300 (EP300), a lysine acetyltransferase [68]. As a result, increased autophagy was observed. 

In human fibroblasts, another mechanism of spermidine-induced mitophagy was observed. Exposure to 50 μM of spermidine was shown to cause mitochondrial depolarization and a ROS burst, which contributed to activation of the protein kinase Ataxia-telangiectasia mutated (ATM) protein [64]. Apart from its role in DNA damage response, ATM also has a function in redox sensing. Activation of ATM contributed to PTEN-induced putative kinase 1 (PINK1) stabilization on the outer membranes of damaged or non-functional mitochondria, which causes translocation of the E3 ubiquitin ligase Parkin (PRKN) to these mitochondria [64]. Ultimately, this leads to the removal of these non-functional mitochondria via mitophagy [64], resulting in improved cell survival.

### 5.3. Polyamines Serve as Energy Scource for Enterocytes

The essential role of polyamines for metabolic function is not restricted to their role in protein transcription or mitochondrial integrity. On a more general metabolic level, putrescine was shown to serve as a direct energy source for intestinal cells. Using labeled putrescine, rat enterocytes were shown to take up putrescine from the lumen [70]. Once taken up, putrescine was in part converted to succinate, and this can be used as a fuel for oxidative phosphorylation (Figure 3) [70]. Although the conversion of putrescine to succinate is not high in normal situations, conversion can greatly increase under certain conditions. A fasting period was found to significantly enhance the conversion of putrescine to succinate in these rats, which remained elevated even 12 h after refeeding [70]. 

### 5.4. Polyamines Influence Metabolism by Depleting Acetyl-CoA Levels

A more indirect way in which polyamines impact metabolism is linked to their dependency on acetyl-CoA as a cofactor for SSAT1 in polyamine acetylation. Polyamine breakdown and export, is for a large part, dependent on the acetylation of the ‘higher’ polyamines by the enzyme SSAT1, in order remove them faster and more efficiently, where acetyl-CoA is the acetyl-donor [25]. Interestingly, acetyl-CoA levels were shown to be depleted upon overexpression of SSAT1, both in vitro and in vivo [71,72,73]. Increased acetylation of polyamines led to induction of ODC1 expression, thus changing overall flux through the polyamine metabolic pathways [73]. Overexpression of SSAT1 in mice led to reduced acetyl-CoA levels in adipose tissue due to the increased acetylation of polyamines, which also correlated with a lean phenotype of the mice [73]. The lean phenotype coincided with increased palmitate and glucose oxidation in the liver [73]. It is likely that increased oxidation is needed to maintain an adequate acetyl-CoA supply. Thus, increased intracellular polyamine levels may deplete cellular acetyl-CoA levels, which may be detrimental if no increase of substrate oxidation can be achieved.

### 5.5. Polyamines Are Transported into Mitochondria and Influence Oxidation

Polyamines also influence metabolic outputs by directly altering mitochondrial functioning. Using isolated mitochondria, it was shown that the mitochondrial matrix can contain high levels of polyamines (around 500 µM for spermine and spermidine, and around 30 µM for putrescine) [74], despite the lack of polyamine biosynthetic pathways within mitochondria. Polyamines within mitochondria, therefore, suggest the presence of a mitochondrial polyamine carrier exists. Indeed, two ATP-dependent mitochondrial carriers have been found for polyamines, termed S1 and S2 (Figure 3) [75]. Spermine and spermidine can both bind to and be transported into mitochondria by each of these carriers, while putrescine can only bind to one. Again using isolated mitochondria, polyamine uptake was found to be dependent on membrane potential, as was demonstrated by Grancara et al. [76] for spermine, and for all four polyamines by Toninello el al. [77]. Grancara et al. [76] found that an increase in the membrane potential from 150 to 180 mV led to a four-fold increase in spermine uptake. Moreover, addition of FCCP, which collapses the membrane potential, caused efflux of spermine [76,78]. Interestingly, spermine constantly cycled over the mitochondrial membrane. In a normal state, with active ATP production, spermine was transported back and forth over the membrane together with ADP and phosphate, which is mediated by adenine nucleotide translocase (ANT) [76]. However, in a state where ATP synthesis was blocked, either through absence of ADP or by inhibition of ATP synthase with oligomycin, spermine efflux is blocked [76]. Spermine was also shown to have a restorative effect on oxidative functions of isolated aged mitochondria [66,79] and it stimulated ATPase activity in bovine heart sub-mitochondrial particles, although it was not mechanistically investigated how this was achieved [80]. Interestingly, spermidine and putrescine exhibited a slightly inhibitory effect on ATPase activity in these sub-mitochondrial particles [80]. 

Although it is unclear what the precise role of polyamines is in mitochondrial physiology, spermine was shown to regulate calcium transport into mitochondria [81]. The calcium-sensitive pyruvate dehydrogenase complex is important for controlling pyruvate influx and subsequent conversion of pyruvate to acetyl-CoA in the mitochondria. Pezzato et al. [78] showed that spermine increased mitochondrial metabolic rate, as seen by a dose-dependent increase in CO_2_ production from pyruvate in isolated rat liver mitochondria upon exposure to spermine, with a maximum increase observed with 0.5–1 mM of spermine. The authors [78] showed that spermine could influence the pyruvate dehydrogenase complex through increased calcium levels within mitochondria. In addition, spermine had a direct effect on the E_1α_-subunit of the complex (Figure 3). Spermine concentrations of up to 0.5 mM led to the highest increase in CO_2_ production, resulting from dephosphorylation of the E_1α_-subunit. Higher concentrations of spermine led to a gradual re-phosphorylation, which corresponded with a slight decrease in CO_2_ production. To exclude possible confounding effects of Ca^2+^ signaling, these experiments were carried out in the presence of ethylene glycol tetraacetic acid (EGTA), which chelates calcium from the environment. Thus, the observed increase in metabolic rate at low spermine levels and decrease at high spermine levels, was shown to be a direct effect of spermine itself, independent of intra-mitochondrial calcium concentration [78]. 

### 5.6. Polyamine Catabolism Leads to Toxic By-Product Formation

In order for a cell to properly regulate the intracellular polyamine levels, polyamines can be oxidized by the amine oxidases polyamine oxidase (PAOX) and spermine/spermidine oxidase (SMOX). These enzymes produce the toxic aldehydes 3-acetoamidopropanal and 3-aminopropanal respectively as by-products during the catabolism of polyamines, as well as H_2_O_2_ [82]. The aldehydes formed by these two oxidases can be converted into acrolein, which is highly toxic. These compounds together may lead to oxidative damage to protein and DNA, which could contribute to cell death [83]. Multiple amine oxidases have also been identified in mitochondria, and oxidation of the polyamines spermine, spermidine and putrescine, together with their acetylated products, was shown to occur within mitochondria (Figure 3) [84]. Local oxidation of polyamines results in production of H_2_O_2_ and aldehydes, which may have adverse effects on mitochondrial functioning.

### 5.7. Polyamines Regulate Formation of the Mitochondrial Permeability Transition Pore

Polyamines play a role in opening of the so called mitochondrial permeability transition pore (MPTP), a non-specific pore that allows bidirectional traffic of metabolites and inorganic compounds over the mitochondrial membrane with a size of up to 1.5 kDa (Figure 3) [85]. It is suggested that the pore has two conformations: a low-conductance state, which is present in normal cell functioning, and a high-conductance mode, which is thought to be one of the main causes of cell death [85]. Although it is still not completely known which proteins make up the MPTP, a polyanion consisting of ten to hundreds of linked phosphate groups (polyphosphate, or PolyP) seems to be essential for its assembly. Spermine was found to selectively inhibit the high-conductance state of the pore, while maintaining its low-conductance form [85]. The proposed mechanism was that spermine could bind to polyphosphate, thereby inhibiting the induction of MPTP opening [85]. Another study found that spermine could also have direct anti-oxidant capacities at a concentration of 100 μM, which may contribute to its role in preventing the complete opening of the MPTP [86]. Interestingly, the induction of the MPTP by oxidative stress seemed to be tissue specific, since rat brain-derived mitochondria were resistant to oxidative stress, whereas rat liver-derived mitochondria succumbed to the stress [87]. 

The impact of polyamines on MPTP was shown to be relevant in models of ischeamia/reperfusion injury. Upon ischemia/reperfusion injury of heart muscles, ODC1 was found to be upregulated, thereby elevating the intracellular polyamine content [88]. This was shown to have a positive effect on survival and recovery [88]. In addition, exogenous administration of spermine to rats prior to ischemia/reperfusion was also found to protect heart tissue from injury [89]. Induction of AKT and MAPK1/3 (Erk2/1) pathways, which are known to have cardio-protective effects in ischemia/reperfusion, could upregulate ODC1. It was proposed that the resulting higher polyamine levels prevented the (complete) opening of the MPTP pore, thereby preventing injury to heart tissue (Figure 3, regulation indicated in red arrows) [88]. In addition, calcium overload was shown to be prevented by exogenous administration of spermine [89]. 

This mechanism may also play a role in intestinal mitochondrial membrane integrity. In the small intestine, polyamine metabolism plays a role in maintaining mitochondrial membrane integrity along the crypt–villus axis. In crypts, the activity of ODC1 was found to be lower compared to villus cells [90]. Since the villus cells do not proliferate, and thus have a lesser need for polyamines, this may seem counterintuitive. However, villus cells have higher respiration rates compared to cells at the bottom of the crypt [90,91]. Perhaps this is because villus cells have an increased energy demand due to active transport of nutrients from the gut lumen. Madsen et al. found that blocking ODC1 activity in rats using α-difluoromethylornithine (DMFO) decreased mitochondrial respiration of intestinal cells, and supplementation of spermine was able to reverse this phenotype [90]. Inhibition of ODC1 was shown to specifically disrupt mitochondrial morphology, without damaging the ultrastructure of the cells. Although the mechanism is not clear, ODC1 thus seems to be crucial also in intestinal cells to maintain mitochondrial respiration and membrane integrity [90], the latter possibly by MPTP opening. 

## 6. Polyamines as Regulators of Intestinal Physiology 

### 6.1. Polyamines Regulate Intestinal Barrier Integrity

The human intestine has a rapid turnover rate, and is thus effectively in a constantly proliferating state [50]. This high level of proliferation coincides with a continuous polyamine requirement to facilitate protein translation. In mice and rats, the proliferative zone of small intestinal and colonic crypts, where the differentiating cells are located, were shown to have especially high polyamine concentrations [92]. The more differentiated cells at the top of the villi had lower intracellular polyamine concentrations [92]. On the other hand, no differences in polyamine levels between villi and crypt cells in isolated human colon cells were found [29]. Although it is unclear whether polyamine levels differ between crypt and villus cells, it is known that they regulate intestinal physiology in various ways, including maintenance of the intestinal barrier function. The intestinal epithelial barrier is maintained by tight junction proteins that form and seal the intestinal barrier. Decreased polyamine levels were shown to result in impaired intestinal barrier integrity [93,94]. Several polyamine-mediated mechanisms regulating the gut barrier have been discovered. On a mRNA transcription level, polyamines were shown to be important regulators of the transcription factor MYC. MYC controls expression of E-cadherin, also known as CDH1, a cell-cell adhesion protein [93]. Polyamines also influence E-cadherin expression through a different mechanism. E-cadherin is sensitive to Ca^2+^-levels. In small intestinal epithelial IEC-6 cells, polyamine depletion caused a decrease in cytosolic Ca^2+^ concentrations [95]. Restoration of intracellular Ca^2+^ levels restored E-cadherin expression to normal levels [95]. Also in IEC-6 cells, the tight junction proteins 1 and 2 (TJP1,2), Occludin (OCLN), Claudin 2 and 3 (CLDN2,3) and E-cadherin were shown to be downregulated in the absence of polyamines [94]. For OCLN, polyamines were found to stabilize the mRNA transcripts. In the absence of polyamines, the half-life of OCLN mRNA was ~75 min, while presence of polyamines increased this to 120 min [94]. 

### 6.2. Polyamines Stimulate Gut Development and Longevity

Polyamines also play a role in gut development in early life. Both human and animal breast milk contain polyamines. Interestingly, the concentration of polyamines were significantly higher in human breast milk of mothers feeding pre-term babies than babies born at term [96]. Perhaps this is a way to stimulate gut development of these young babies. Supplementation of polyamines to milk-formulas of piglets was shown to increase expression of maturation markers in the small intestine [97,98]. In addition, spermine supplementation improved the crypt-to-villus ratio at weaning when administered during the suckling phase in piglets [99]. Crypt-to-villus ratio is often used as a way to assess intestinal morphology and function [100], with 3:1 is considered an optimal ratio for the small intestine. It is interesting to note is that in animal husbandry, antibiotics were previously used in low doses as an in-feed growth promotor during the post-weaning period. This practice was shown to increase polyamine levels, especially that of putrescine, spermidine and cadaverine [101,102]. By stimulating polyamine levels, the antibiotic treatment may have contributed to maturation of the gut of young piglets. This may have contributed to pig survival, since the intestine of newly weaned piglets is often not fully developed [103]. Although the mechanisms are not well investigated, polyamine supplementation through the diet may well have enabled optimal protein translation in the immature gut. 

Additionally, in the mature gut, there is evidence that luminal polyamines continue to play an important role. In humans it has been shown that polyamine concentrations decline in the fecal content with age [13]. Administration of 4 mM of spermidine in the medium of yeast, or 0.3 and 3 mM in the drinking water of mice, was shown to increase longevity [69]. The increased spermidine content in the intestine was related to hypoacetylation of histone 3 in enterocytes, which during ageing may become hyperacetylated. Increased spermidine-induced acetylation of histone 3 led to upregulation of autophagy-related gene expression, which contributed to increased longevity [69]. The mechanisms of autophagy induction by spermidine described above contribute to increased longevity, as a result of the autophagy-mediated removal of dysfunctional organelles, cells or proteins, preventing cell damage and ultimately cell death [65,69,104]. Interestingly, naked mole-rats, rodents that have a 10 times longer life-expectancy than mice of the same size, were shown to maintain polyamine levels even during ageing [105].

### 6.3. Do luminal Polyamine Levels Influence Intracellular Concentrations in Enterocytes?

Within the gut, polyamines play a variety of roles; be it in cell proliferation, metabolic regulation or physiological functions of the intestine. Gut polyamines can originate from endogenous production within enterocytes, from the diet, or through bacterial fermentation of protein. In the small intestine, dietary polyamines are main source, and uptake from the lumen is a rapid process [106]. In the colon, bacterial fermentation of protein is the main source of polyamines, and the more protein that enters the colon through increased dietary consumption, the more polyamines produced by bacteria [107].

An important question that arises is whether the increased polyamine levels produced by the microbiota in the colon actually influence colonic tissue polyamine levels or gut physiology. From cell culture experiments, we know that polyamines present in the medium do end up in the cells. For example, exposure of human HCT-116 colon cancer cells to putrescine, spermidine and spermine increases the intracellular concentration of the polyamines in a dose-dependent manner [108]. Similarly, when human HT-29 colon cancer cells were exposed to the acetylated polyamine N^1^,N^12^-diacetylspermine, which is hypothesized to play a role in colon carcinogenesis, this compound could be detected intracellularly 24 h after dosing [109]. In the small intestine of rats, putrescine was also shown to be taken up from the lumen, and could even be used as an energy source [70]. Administration of polyamines through drinking water of piglets was also shown to improve gut maturation of the small intestine [97,99]. In suckling rats, long-term feeding of a polyamine deficient diet resulted in intestinal hypoplasia, both in the small and the large intestine, although no differences in mucosal polyamine concentrations were observed [110]. These studies indicate that there may at least be an interaction between luminal polyamine concentrations and enterocytes. However, no mechanisms are known as to how luminal polyamines influence the intestinal tissue. Whether in vivo increases in luminal polyamine concentrations also increase the colonic tissue content thus remains to be seen. When comparing the levels of polyamines in mid-colonic tissues of germ-free and former germ-free mice, Matsumoto et al. [111] observed no increase in the levels of intracellular polyamines. At the same time, the concentrations of putrescine in colon feces was increased seventeen times, and spermidine concentrations were two times higher, in ex-germ free mice [111]. Thus, although colonic feces concentrations were markedly increased, no reflection of this increase was observed in colonic tissue. A soy protein-based diet was shown to increase luminal polyamine levels in the colon of pigs, coinciding with increased red blood cell polyamine content, but this did not result in increased colonic proliferation or ODC1 expression [21]. Although colonic uptake of luminal putrescine has been observed, this was under conditions of polyamine synthesis inhibition through treatment with DFMO [108]. Thus, it remains unclear whether increased luminal polyamine concentrations result in increased colonic tissue levels under physiological conditions. 

## 7. Conclusions

Increased protein intake, shown to be effective as a means to improve weight-loss or sports performance, leads to increased protein fermentation within the colon. As a consequence, polyamine levels in the lumen of the colon increase. Even though it is known that polyamines play a role in a plethora of cellular functions, much less is known about how luminal polyamines can affect colonic physiology. Perhaps the tight regulation of polyamine levels within the colonocytes is so effective that an increase in luminal polyamine levels will not affect tissue homeostasis. However, since there have been few investigations of the effect of increased luminal polyamines under physiological conditions, no definitive conclusions can be drawn, although it is clear that the outcome will be tissue and concentration-dependent. Studies that specifically explore the interactions between luminal polyamine levels in the colon, EIF5A hypusination, gut proliferation and metabolic function could perhaps further our understanding of how luminal polyamines can influence or interact with normal colonic physiology when high-protein diets are consumed.
